# A Multimetric, Map-Aware Routing Protocol for VANETs in Urban Areas

**DOI:** 10.3390/s140202199

**Published:** 2014-01-28

**Authors:** Carolina Tripp-Barba, Luis Urquiza-Aguiar, Mónica Aguilar Igartua, David Rebollo-Monedero, Luis J. de la Cruz Llopis, Ahmad Mohamad Mezher, José Alfonso Aguilar-Calderón

**Affiliations:** 1 Department of Telematic Engineering, Universitat Politécnica de Catalunya (UPC), C/ Jordi Girona 1-3, Barcelona 08034, Spain; E-Mails: luis.urquiza@entel.upc.edu (L.U.-A.); maguilar@entel.upc.edu (M.A.I.); david.rebollo@entel.upc.edu (D.R.-M.); luis.delacruz@entel.upc.edu (L.J.C.L.); ahmad.mezher@entel.upc.edu (A.M.M.); 2 Faculty of Informatics, Autonomic University of Sinaloa (UAS), De los Deportes Avenue and Leonismo Internacional s/n, Mazatlan 82107, Mexico; E-Mail: ja.aguilar@uas.edu.mx

**Keywords:** vehicular *ad hoc* networks, multi-metric forwarding decisions, geographic routing protocol

## Abstract

In recent years, the general interest in routing for vehicular *ad hoc* networks (VANETs) has increased notably. Many proposals have been presented to improve the behavior of the routing decisions in these very changeable networks. In this paper, we propose a new routing protocol for VANETs that uses four different metrics. which are the distance to destination, the vehicles' density, the vehicles' trajectory and the available bandwidth, making use of the information retrieved by the sensors of the vehicle, in order to make forwarding decisions, minimizing packet losses and packet delay. Through simulation, we compare our proposal to other protocols, such as AODV (Ad hoc On-Demand Distance Vector), GPSR (Greedy Perimeter Stateless Routing), I-GPSR (Improvement GPSR) and to our previous proposal, GBSR-B (Greedy Buffer Stateless Routing Building-aware). Besides, we present a performance evaluation of the individual importance of each metric to make forwarding decisions. Experimental results show that our proposed forwarding decision outperforms existing solutions in terms of packet delivery.

## 1. Introduction

Vehicular *ad hoc* networks (VANETs) [1,2] are an emerging area of wireless networking that facilitate ubiquitous connectivity among smart vehicles through vehicle-to-vehicle (V2V) communications and between vehicles and the city or the road infrastructure through vehicle-to-roadside (V2R) communications. This emerging technology field aims to improve the safety of passengers, alleviate the traffic flow, reduce pollution and enable in-vehicle entertainment applications for passengers. Safety applications can reduce accidents by providing traffic information to drivers, such as collision warning, road surface conditions or the state of the traffic flow. Moreover, passengers could use the available infrastructure of the city to connect to the Internet for entertainment applications [3].

The growing interest in this technology is an incentive for car manufacturers, the research community and governments who, day after day, increase their efforts towards creating a standardized platform for vehicular communications. The unique characteristics and some special requirements of VANETs generate different challenges for the research community. To address these challenges in both safety and comfort-oriented applications, there is a pressing need to develop new routing protocols specially designed for this kind of network, which provide a good performance, either under sparse or dense traffic conditions. This work proposes a new routing protocol for VANETs, which considers several metrics to select the best next forwarding node for each packet in each step towards its destination. Simulation results show the benefits of our protocol compared to other proposals under different network conditions.

## 2. Related Work

Traditional traffic management systems are based on centralized infrastructures in which cameras and sensors placed along the roadside collect information about vehicle density and the traffic state. These data are sent to a central unit that processes them and makes appropriate decisions. This type of framework is very costly in terms of deployment and is characterized by the long time needed to process information.

The rapid development of wireless communication has raised the interest for car manufacturers, the research community and governments, making a new decentralized architecture based on vehicle-to-vehicle communications. Vehicular *ad hoc* networks are a specific type of mobile *ad hoc* network (MANET) [1] in which the nodes are vehicles. The road-constrained characteristics of these networks, the high mobility of the vehicles, their unbounded power source and the presence of roadside wireless infrastructures make VANETs an important research topic. The main purpose of VANETs is to be a platform that can support intelligent inter-vehicle communication, improving traffic safety, although they are also useful for traffic management and other services, e.g., Internet access.

The presence of city infrastructure in emerging smart cities has an important impact on urban communications. In some applications, one must find a route to the closest access point (AP), e.g., connecting to the Internet. In a city with advanced wireless infrastructure deployed, it would take only a few hops to reach the nearest AP under day-traffic conditions. Roadside access points should be placed in special locations, such as on traffic lights, since they are well positioned to act as traffic routers. They already form a traffic grid, usually located where traffic is most intense, and are equipped with a power supply and directly maintained by local municipalities. For instance, [4] proposes a priority intersection control scheme in a self-organized manner, so that smart traffic lights detect the presence of emergency vehicles and assign them a priority at intersections. The goal is to provide a “green-wave” signal display for the emergency vehicles seeking to avoid accidents.

Several proposals of routing protocols for VANETs have been presented. Research works claim that the best routing protocols for VANETs are those that use geographic routing, which are based on the knowledge of the instantaneous locations of nodes [5].

GPSR (greedy perimeter stateless routing) [6] is a well-known geographic routing protocol specially designed for VANETs. It forwards packets to the neighbor node that is closest to destination following a hop-by-hop scheme. GPSR uses two different techniques to forward packets: *greedy forwarding*, which is used by default, and *perimeter forwarding*, which is used whenever greedy forwarding cannot be used. Since nodes require knowing their neighbors' positions, they periodically transmit a hello message containing their own identifier (e.g., IP address) and their position. Several proposals that improve GPSR have been presented in the literature. The authors in [7] proposed movement prediction-based routing (MOPR), which improves the routing process of GPSR by selecting the most stable route in terms of lifetime with respect to the movement of vehicles. In [8], an algorithm was proposed that modifies GPSR and exploits information about movement to improve the next forwarding node decision. The authors use information about position, moving direction and speed to make routing decisions. The proposed protocol was compared to GPSR in a highway setting, showing improvement. Advanced Greedy Forwarding (AGF) [9] improves the performance of GPSR, since its forwarding technique is more fault-tolerant than the traditional greedy forwarding.

Recently, some proposals of routing protocols for VANETs that include several metrics have been presented. The use of alternative metrics in VANETs in addition to the classic distance has shown notable benefits. For instance, the trajectory of the vehicles is used in [7,10]. The authors in [11] propose the Improvement GPSR routing protocol (I-GPSR), which incorporates distance, vehicle density, moving direction and vehicle speed to make packet forwarding decisions. A new data forwarding mechanism, which uses a store-carry-forward scheme besides the position information and moving direction of the nodes, was presented in [10].

Nevertheless, none of the existent proposals takes the available bandwidth into account as a metric to make forwarding decisions. The reason could be that obtaining a fairly simple, but accurate, model to calculate the available bandwidth in VANETs is a difficult task. Our proposal includes several metrics to optimize the selection of the next forwarding node in a geographic-based protocol. These metrics are vehicle density, trajectory, distance to destination and available bandwidth. The trajectory of a node consists of its moving direction and speed, which are used to estimate how fast a node gets near to or goes away from a destination. Then, we weight those four metrics into a single multimetric score. Furthermore, we evaluate the performance of each single metric compared to the global multimetric value and also to some combination of them.

## 3. Multimetric Map-Aware Routing Protocol (MMMR)

### 3.1. Motivation

VANET nodes are vehicles that move along roads, potentially at a high speed. These vehicles follow transit rules, respect the direction of the streets, traffic lights and also the presence of buildings and other vehicles. The vehicle density in VANETs constantly changes depending on the area and the time of the day, so it is difficult to establish and maintain end-to-end communication paths between sources and destinations, as is traditionally done in MANETs. Several routing protocols based on geographic information have specially been proposed for VANETs. Nonetheless, a proper data forwarding mechanism is still needed to cope with the special constraints of VANETs, e.g., the high nodes' speed, the dynamic network topology and the variable nodes' density.

We propose a new routing protocol for VANETs in urban scenarios that we call the multimetric map-aware routing protocol (MMMR). MMMR seeks to improve the decision of the next forwarding node based on four routing metrics, which are the distance to destination, the vehicles' density, the vehicles' trajectory and the available bandwidth. We weight the four metrics to finally obtain a multimetric value for every neighbor node that is a candidate as the next forwarding node. The scheme is self-configured and able to adapt to the changing vehicles' density in real time.

In our proposal, we can distinguish five processes: map-aware capability, use of a local buffer, signaling, the evaluation of metrics and the forwarding decision. We explain each one in the following.

### 3.2. Map-Aware Capability and Use of a Local Buffer

An important issue that one should face in the design of a routing protocol for VANETs in urban scenarios is the presence of the buildings, trees and other obstacles usually found in cities. Greedy forwarding in GPSR is often restricted,because direct communication between nodes may not exist, due to an obstacle. We include a map-aware capability, which focuses on two important enhancements: (a) it takes into account the presence of buildings in the decision of the next forwarding node; and (b) it uses a local buffer to temporarily store packets when no forwarding node was found.

The forwarding decision included in MMMR uses the same criteria as the original GPSR [6]. It consists in choosing the neighbor located nearest to a destination. Besides, our proposal tackles the important issue of deciding which neighbors are actually reachable. This feature is of paramount importance to determine if a neighbor in the list could be a good forwarding node. The accurate information about the current position of a neighbor has a strong impact on the routing performance, because knowing this information makes the node aware of which neighbors are reliable to act as the next forwarding nodes. At the same time, it avoids sending packets to an unreachable node, because it is, for instance, behind a building. This feature makes vehicles be map-aware, since they avoid sending packets to nodes behind the walls of the buildings in the city. The presence of obstacles, such as buildings, are tackled using vehicular location. If the straight line between the current vehicle position and the next vehicle position is blocked by any rectangular area representing a building, then that node behind a building cannot be chosen as the next forwarding node. Otherwise, packets sent to that node would be lost.

Furthermore, an important drawback of GPSR is the implementation of *perimeter forwarding*, because it is not clear when the algorithm switches its mode to greedy forwarding again. The main problem of GPSR is the use of outdated information to select the next forwarding node. It is possible to find inconsistencies in the neighbor tables or in destination node's location set in the packet. The neighbor table problem is to select a node that is out of range, resulting in packet loss. This happen often because often, the nearest neighbor to the destination node is also the farthest neighbor. Due to destination node's location included in the packet, the header is never updated by any forwarding node; packets with high a delay will never arrive to the destination node and will get lost in a location where the destination is no longer. In addition, mobility can induce routing loops while using the *perimeter mode* [12]. To avoid these problems caused by perimeter forwarding, our proposal stores packets in a local buffer of the current relaying node when there is no neighbor that satisfies all the requirements needed to be a next forwarding node. If at least one of the two conditions (i.e., being actually a reachable neighbor and being closer than the current carrier node to the destination) required to be the next forwarding node is not satisfied, then packets are stored according to the FCFS (First-Come, First-Served) scheme in a local buffer instead of being discarded. If the buffer gets full, packets will be dropped. The nodes periodically look for a neighbor that can satisfy the requirements to be the next forwarding node. Every period of time (1 s), the node looks for a new node candidate. After previous simulations analysis, it was concluded that this period of time is frequent enough to detect quickly any topology change. If a candidate fulfills the requirements, the stored packets are forwarded to that node. In our case, the impact of out of order is not remarkable, due to the kind of services that we use, such as the report of accidents, traffic or environmental information obtained by the car sensors.

We use the LOS (line of sight) criteria, because our evaluation scenario is a city, i.e., an urban zone with walls and buildings. Besides, the evaluated network is a VANET (nodes are vehicles). Thus, in this case, the more important and restrictive point to take into account is the line of sight of the nodes in the network. Due to that, in the moment the forwarding decision is made, we act in a conservative way, considering only as candidates to be the next forwarding node those nodes that actually can receive the packet, because they are in the LOS of the current carrying node.

Concluding, three conditions must be verified for a node to be chosen as the next forwarding node, i.e., being in the coverage range, in LOS (line of sight) and nearer to a destination than the current node. If the candidate fulfills all three requirements, packets are forwarded to it; otherwise, packets are stored in the local buffer of the current carrier node.

In addition to these two improvements, the decision of the next forwarding node is based on the combination of four metrics, which are detailed in Section 3.4.

### 3.3. Signaling

MMMR, as most geographic routing protocols, requires nodes to periodically send signaling messages announcing their presence to the neighbors in transmission range. To obtain precise location information about each node without introducing new signaling messages, we use a new format of the existing hello messages (HM) to exchange information between neighbors. The format of hello messages used by MMMR is presented in Table 1, and they include the following fields:

- **ID:** A four-byte field with the identifier of each node.
- **Position:** This field is divided into 32 bits for latitude (*l_x_*) and 32 bits for longitude (*l_y_*), which represent the geographic position of each node. Each set of 32 bits is also divided into one bit for the direction (north or south for longitude; east or west for latitude), eight bits for the grades, six bits for the minutes and 17 bits for the seconds.
- **Velocity:** A two-byte field that uses one byte for speed (m/s) in the *x*-axis (*v_x_*) and one byte for speed in the *y*-axis (*v_y_*). Each node calculates its own speed from two consecutive position points, (*x*_1_,*y*_1_) and (*x*_2_, *y*_2_), taken at times *t*_1_ and *t*_2_:
  (1) $$\begin{array}{cc} v_{x}=\frac{x_{2}-x_{1}}{t_{2}-t_{1}}, & v_{y}=\frac{y_{2}-y_{1}}{t_{2}-t_{1}} \end{array}$$
- **Antenna sensing** (*S*): One byte to express the antenna sensing in the power ratio in decibels (dBm) used by the node. A signal power below the antenna sensing is not detectable.
- **Idle time** (*t_idle_*): Each node calculates the units of time that it spends without sending nor receiving data since the last HM sent. This value is represented in one byte. That is, *t_idle_* measures the amount of time the node is idle between two consecutive hello messages.
- **Density** (*ρ*): One byte to represent the number of neighbors within the transmission range at the moment of sending the current hello message.

When a node receives a hello message from a neighbor in transmission range, the node stores the reception time and updates its neighbor list with all the values shown in Table 1. This is done following the conditions shown in Algorithm 1. If a hello message is received from a neighbor already registered in the neighbor list, the information of this neighbor is just updated (Lines 1, 2 from Algorithm 1). To keep the list of neighbors updated and to use only nodes that actually are in transmission range, nodes remain in a neighbors list for twice the interval between consecutive hello messages.

In addition, we were careful to check whether a node was located too close to the transmission range before considering it as a neighbor. When a node receives a hello message from a new neighbor (a neighbor not yet registered in the list), it has first to check if this is a stable node before adding it in the neighbor list. This means that hello messages have to be received with a power higher than the antenna sensing plus a security margin (Lines 4 to 8). We set this security margin to 1 dB, since we observed from simulation results that this was a proper value. If this condition is not fulfilled, that neighbor will not be included in the neighbor list (Line 7), since this node probably is around the border line of the transmission range.


**Algorithm 1** Updating the neighbor list.
**Require:** A new hello message received with these parameters: ID, *l_x_, l_y_, v_x_, v_y_, S, t_idle_*, *ρ*.1:**if** (The neighbor is already in the neighbor list) **then**2: Update neighbor information3:**else**4: **if** (Reception power ≥ antenna sensing + 1 dB) **then**5:  Add node in the neighbor list6: **else**7:  Ignore hello message8: **end if**9:**end if**


The sending period of hello messages could be higher to obtain more accuracy in the composition of the list of neighbors, although a higher signaling traffic could produce an increase in packet collisions. By default, the sending period of the HM is set to 1 s.

The list of neighbors includes the data sent in hello messages (see Table 1) and the data shown in Table 2. For each neighbor, *i*, we store the reception time when the last hello message arrived (the *reception time of the last HM* in Table 2). This is done to estimate the future position of that neighbor node. Furthermore, we store the moment when the first hello message arrived (the *reception time of the first HM*)and the total number of hello messages received (*No. HM*). These values will be used to estimate the available bandwidth using a metric explained in the next section.

### 3.4. Metrics Evaluation

In this section, we detail each one of the four metrics included in our multimetric algorithm MMMR, which is a geographic routing protocol based on hop-by-hop forwarding decisions. The use of these metrics is a way to improve the choice of the next forwarding node. The four metrics considered are the distance to a destination, the trajectory of the vehicles, the nodes' density and the available bandwidth. They are described below.

**Distance:** The typical goal of most geographic routing protocols is to send packets hop-by-hop to their destination, so that the next forwarding hop is the neighbor that is closest to the destination. These kind of protocols are based on the knowledge of geographic information of every node in the scenario. Each vehicle knows its own position and also the position of the destination. Therefore, it is usually assumed that the position of the packet's destination and the positions of the next hop candidates are sufficient to make proper forwarding decisions. We also use the distance (*d*) to the destination as a metric to evaluate each neighbor in our proposal. We obtain this information from the fact that senders know the destination position (*x_D_*, *y_D_*) and also each neighbor includes in the hello messages its own position. With the position (*x_i_*, *y_i_*) of each neighbor, *i*, we can obtain its Euclidean distance (*d_i_*) to the destination (*D*) (see Equation (2)):

(2) $$d_{i}\left(\left(x_{i},y_{i}),(x_{D},y_{D}\right)\right)=\left\|{\vec{x}}_{i}-{\vec{x}}_{D}\right\|=\sqrt{{\left(x_{i}-x_{D}\right)}^{2}+{\left(y_{i}-y_{D}\right)}^{2}}$$

**Figure 1.**
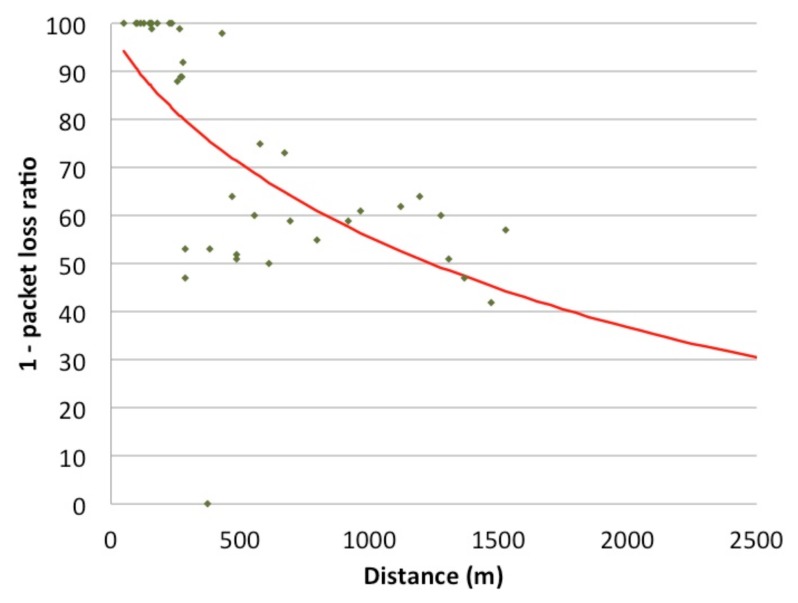
The relation between the distance to the destination and packet losses.

To compute the metric based on this distance, *u*_1,_*_i_*, we use Expression (3), where *d_i_* is the previous Euclidean distance and *d_ref_* is a reference distance, above which losses start to increase notably faster. This *d_ref_* is obtained from simulations where we evaluated the performance of a fixed node set in different distances from the AP. The results are presented in Figure 1, where we can see that *d_ref_* is around 2,000 m in our urban scenario. Notice that for a transmission range of 250 m, such a path of around 2,000 m would take around eight hops. *α* is an attenuation factor that equals 0.77, obtained after a mathematical regression using the results of the simulations mentioned before and shown in Figure 1. This value, represented in Figure 1, measures the utility of the metric of the distance. Consequently, low distances to the destination obtain a high benefit, while long distances to the destination obtain low profits. The main settings used in this evaluation are presented in Table 3.

In this case, the shorter value of the distance, *d_i_*, the better that node *i* will be classified, because we prefer a neighbor as close as possible to the destination. This way, Equation (3) tends to one when the distance to destination *d_i_* tends to zero. We selected a negative exponential function to penalize drastically those neighbors with bad values in this metric. This means that the best forwarding node will get a score notably higher than the others. This way, we highlight those good candidates, so that they have more chances to be selected as the next forwarding nodes.

(3) $$u_{1,i}(d_{i})=e^{-{\left(\frac{d_{i}}{d_{\text{ref}}}\right)}^{\alpha }}$$

**Trajectory:** A communication link in a VANET remains operative for a short time, due to the high speed of the vehicles. If a geographic protocol does not consider the moving direction of the nodes, the current node could make wrong forwarding decisions based only on the distance and send packets to vehicles that were actually going away from the destination. Therefore, packet losses could increase. For that reason, taking into account the moving direction of vehicles is an important feature in VANETs. Hence, we decided to design a metric to obtain an accurate measure of the trajectory of the vehicles in VANETs.

**Figure 2.**
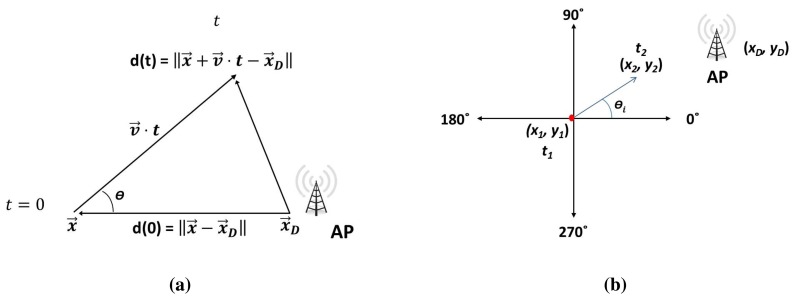
Trajectory towards the access point (AP) from two consecutive geographic positions.

We define the trajectory metric of node *i* as a comparison of the metric of the future distance to the destination in time *t* of that node, *i.e.*, *u*_1,_*_i_*(*d*(*t*)), with respect to the metric of the current distance to the destination in *t* = 0, *i.e.*, *u*_1,_*_i_*(*d*(0)). We can see the result in Equation (4) and a drawing in Figure 2a. That is, we prefer those forwarding nodes that get to the destination sooner. Therefore, consecutive values of the metric of the distance (*i.e.*, *u*_1,_*_i_*(*d_i_*), for a moving node will grow throughout time, given that the node gets closer to the destination.

According to Equation (5) and Figure 2a, the distance to the destination of the node in *t* (*i.e.*, *d*(*t*)) is computed from its current position in *t* = 0 (*i.e.*, *x⃗*), the position of the destination (*i.e.*, *x⃗_D_*) and the average speed, *v⃗_i_*. The average speed, *v⃗_i_*, is computed from two consecutive positions (see Figure 2b). ‖ · ‖ refers to the module function of a vector. The future position in *t* is *x⃗*+ *v⃗ · t*. Using the speed of the node (*i.e.*, *v⃗_i_*), we can give a higher score to nodes that will be closer to the destination sooner (AP). The idea is that with a higher speed, a node may arrive sooner to the destination, given that the distance to the destination decreases.

(4) $$u_{2,i}\left(d(t),d(0)\right)=\frac{u_{1,i}\left(d(t)\right)}{u_{1,i}\left(d(0)\right)}$$

(5) $$d(t)=\left\|\vec{x}+{\vec{v}}_{i}\cdot t-{\vec{x}}_{D}\right\|$$

Substituting the expression of the metric of the distance expressed in Equation (3) into the metric of the trajectory defined in Equation (4), we obtain:

(6) $$u_{2,i}\left(d(t),d(0)\right)=\frac{e^{-{\left(\frac{d(t)}{d_{\text{ref}}}\right)}^{\alpha }}}{e^{-{\left(\frac{d(0)}{d_{\text{ref}}}\right)}^{\alpha }}}=e^{-\frac{1}{d_{\text{ref}}\alpha }\left(d{\left(t\right)}^{\alpha }-d{\left(0\right)}^{\alpha }\right)}$$

Then, we compute the metric of the trajectory, *u*_2,_*_i_*(*d*(*t*), d(*0*)), using Equation (8), where we define Δ*d*(*t*)*^α^* as a measure of the variation of the distance in a time, *t*, to the power of *α* with respect to the current distance in *t* = 0 to the power of *α*.

(7) $$\Delta_{d}{\left(t\right)}^{\alpha }=d{\left(t\right)}^{\alpha }-d{\left(0\right)}^{\alpha }$$

(8) $$u_{2,i}\left(d(t),d(0)\right)=e^{-{\left(\frac{\Delta_{d}(t)}{d_{\text{ref}}}\right)}^{\alpha }}$$

Again, the use of an exponential function helps us to emphasize good nodes compared to the others. A higher qualification in *u*_2,_*_i_*(*d*(*t*), *d*(*0*)), is obtained if the node presents a faster decrease of its distance to the destination.

Next, using a Taylor approximation in the estimation of the future position, *d*(*t*), we obtain Equation (9), where *O*(*t*^2^) represents the upper order terms:

(9) $$d{\left(t\right)}^{\alpha }={d{\left(0\right)}^{\alpha }+\frac{d}{dt}d{\left(t\right)}^{\alpha }|}_{t=0}\cdot t+O(t^{2})$$

To facilitate the calculation, we write Equation (9) in terms of the well-known expression, *d*(*t*)^2^ (*i.e.*, with *α* = 2). This results in the computation of $\frac{d}{dt}{\left(d{\left(t\right)}^{2}\right)}^{\frac{\alpha }{2}}$.

(10) $$\begin{array}{l} d{\left(t\right)}^{2}={\left\|\vec{x}+\vec{v}\cdot t-{\vec{x}}_{D}\right\|}^{2}\\ \;={\left\|\vec{x}-{\vec{x}}_{D}\right\|}^{2}+2\cdot \langle \vec{x}-{\vec{x}}_{D},\vec{v}\rangle \cdot t+{\left\|\vec{v}\right\|}^{2}\cdot t^{2} \end{array}$$

where 〈·, ·〉 refers to the scalar product. Notice that comparing Equation (10) to the Taylor approximation in Equation (9), where *d*(0)*^α^* = *d*(*x⃗*, *x⃗*_*D*_)*^α^* =‖*x⃗*−*x⃗_D_*‖*^α^* (see Equation (2)), we obtain:

(11) $${\frac{d}{dt}d{\left(t\right)}^{2}|}_{t=0}=2\cdot \langle \vec{x}-{\vec{x}}_{D},\vec{v}\rangle$$

Applying the chain rule in $\frac{d}{dt}{\left(d{\left(t\right)}^{2}\right)}^{\frac{\alpha }{2}}$ yields:

(12) $$\frac{d}{dt}d{\left(t\right)}^{\alpha }=\frac{\alpha }{2}\cdot {\left(d{\left(t\right)}^{2}\right)}^{\frac{\alpha }{2}-1}\cdot \frac{d}{dt}d{\left(t\right)}^{2}$$

At *t* = 0, we have *d*(*0*) = *d* and ${\frac{d}{dt}d{\left(t\right)}^{2}|}_{t=0}=2\cdot \langle \vec{x}-{\vec{x}}_{D},\vec{v}\rangle$. Hence,

(13) $${\frac{d}{dt}d{\left(t\right)}^{\alpha }|}_{t=0}=\frac{\alpha }{2}\cdot d^{2\left(\frac{\alpha -2}{2}\right)}\cdot 2\cdot \langle \vec{x}-{\vec{x}}_{D},\vec{v}\rangle$$

Let *θ* be the angle between vectors *x⃗* − *x⃗_D_* and *v⃗* · *t*; see Figure 2a. On account of the fact that 〈*x⃗* − *x⃗_D_*, *v⃗*〉 = *d* · ‖*v⃗*‖ · cos*θ*, we have

(14) $$\begin{array}{cc} {\frac{d}{dt}d{\left(t\right)}^{\alpha }|}_{t=0} & =\alpha \cdot d^{\alpha -2}\cdot d\cdot \left\|\vec{v}\right\|\cdot \cos\theta \\ & \;=\alpha \cdot d^{\alpha -1}\cdot \left\|\vec{v}\right\|\cdot \cos\theta \end{array}$$

Replacing Equation (14) in Equation (9), we obtain:

(15) $$d{\left(t\right)}^{\alpha }≃d^{\alpha }+\alpha \cdot d^{\alpha -1}\cdot \left\|\vec{v}\right\|\cdot \cos\theta \cdot t$$

Next, comparing Equation (15) to Equation (7), we achieve an expression for Δ*_d_*(*t*)*^α^*, *i.e.*, our proposed measure of the variation of the distance in a time, *t*, to the power of *α* with respect to the current distance in *t* = 0 to the power of *α*.

(16) $$\Delta_{d}{\left(t\right)}^{\alpha }≃\alpha \cdot d^{\alpha -1}\cdot \left\|\vec{v}\right\|\cdot \cos\theta \cdot t$$

Equation (15) can be divided into two parts: the current distance, *d*, to the power of *α,* and our measure of the future position, Δ*_d_*(*t*)*^α^*.

Finally, replacing Equation (16) in Equation (8), we obtain an equation for the metric of the trajectory of vehicles, where we set *t* = 1 s as the interval of time to evaluate the trajectory of the vehicle, *i*:

(17) $$u_{2,i}\left(d(t=1),d(t=0)\right)=e^{-\left(\frac{\alpha \cdot d^{\alpha -1}\cdot \left\|\vec{v}\right\|\cdot \cos\theta }{d_{\text{ref}}^{\alpha }}\right)}$$

Due to the exponential behavior of metrics *u*_1,_*_i_*(*d_i_*) and *u*_2,_*_i_*(*d*(*t*), *d*(*0*)), we decided to use exponential functions also to define the metrics of density *u*_3,_*_i_* and available bandwidth *u*_4,_*_i_*. This way, we avoid having an excessive influence of metrics *u*_1,_*_i_* and *u*_2,_*_i_* in the final value of the multimetric score, because these metrics could obtain high values that might affect the final score.

**Density:** This is computed as the number of vehicles in the list of neighbors (*N_neigh_*) of each node at the moment, *t*, of sending the current hello message, divided by the transmission range (*Tx*) of the node. The list of neighbors is composed of vehicles in transmission range that send packets with enough power to be considered stable neighbors. Each node, *i*, computes its current density of nodes *ρ_i_*(*t*) and includes it in the next hello message.

(18) $$\rho_{i}(t)=\frac{N_{\text{neigh}}(t)}{Tx}$$

According to Equation (19), the algorithm gives a higher score (bounded by one) to this metric when the node has a higher value of the density of nodes, *ρ_i_*. Nodes with a denser area in the transmission range will have more possibilities to forward the packet to a better next node. This is true until reaching a maximum traffic density, above which, the too high number of vehicles in the surrounding area of each node increases the frequency of collisions. This maximum traffic density will depend on the specific scenario and configuration parameters and will be determined in each simulation.

We evaluate our proposal in two scenarios with different vehicle densities, to be able to analyze the performance of the next forwarding selection used by our protocol. Our approach to calculate the density metric uses a simple algorithm that gives a higher score to those nodes with higher vehicle densities. Moreover, we leave to the available bandwidth metric the task of dealing with the data congestion problem. For instance, a node with a high vehicle density metric that has a congestion problem (*i.e.*, a low available bandwidth) will be highly rated by the density metric, but will also be penalized by the available bandwidth estimator (ABE) metric. In this way, we face the trade-off between density and congestion.

(19) $$u_{3,i}(t)=e^{-\frac{1}{\rho_{i}(t)}}$$

**Available bandwidth:** Multimedia services (e.g., video streaming) require a given amount of network resources to run correctly. To improve the QoS (Quality of Service) provided with our proposal, we included an estimator of the available bandwidth in VANETs based on a previous approach, called the available bandwidth estimator (ABE) developed in [13]. The ABE algorithm estimates the available bandwidth in a link between two general nodes in IEEE 802.11 networks. We previously evaluated ABE for VANETs in [14] and improved its operation further in [15]. Here, we summarize the ABE proposal, while a full explanation can be found in [13].

Each node estimates its percentage of idle time by sensing the common wireless medium. This value is included in its hello messages. The ABE algorithm uses the idle times of the emitter (*T_s_*) and the receiver (*T_r_*) of a link of capacity *C*. Furthermore, ABE computes the collision probability of the hello messages, named *p_hello_*. The packet collision probability of packets of m bits, named *p_m_*, is derived from the collision probability of the hello messages using Equation (20).

(20) $$p_{m}=f(m)\cdot p_{\text{hello}}$$

The function, *f*(*m*), is used in Equation (20) to estimate the packet collision probability that is included in the ABE algorithm. This *f*(*m*) was obtained in [13] by computing the Lagrange interpolating polynomial, taking pairs of values of packet losses (*p_m_*) and losses of hello messages (*p_hello_*) from simulations. The result was *f*(*m*) = −5.65·10^−9^·*m*^3^ + 11.27·10^−6^·*m*^2^ − 5.58·10^−3^·*m* + 2.19. The additional overhead introduced by the binary exponential backoff mechanism used in IEEE 802.11 networks was computed in [13] using Equation (21).

(21) $$K=\frac{\text{DIFS}+\bar{\text{backoff}}}{T_{m}}$$

where *T_m_* (in s) is the time separating the emission of two consecutive frames, DIFS (Distributed Inter Frame Space) [16] is a fixed interval and $\bar{\text{backoff}}$ is the number of backoff slots decremented on average for a single frame. Finally, a node estimates the available bandwidth, ABE*_i_*, on each neighbor's, *i*', wireless link using Equation (22) [13]. Notice that for us, *s* is the current forwarding node and *r* refers to every neighbor, *i*, with which *s* establishes a link.

(22) $${\text{ABE}}_{i}=(1-K)\cdot (1-p_{m})\cdot T_{s}\cdot T_{r}\cdot C$$

As was done in [13], in this present work, we considered the packet size (m) as a variable, but also, we took into consideration other variables that are determinant in vehicular scenarios: the nodes' density (*N*) and the average speed of the nodes (s). Our goal was to obtain a new function, *f*(*m*, *N*, *s*), to be included in Equation (20) instead of the former *f*(*m*) used in [13]. The routing protocol will be able to make better forwarding decisions to minimize packet losses in VANETs, since *N* and *s* are determinant configuration parameters.

We computed a multiple lineal regression [17] using the software, IBM SPSS 20 [18], with results obtained from simulations, varying the three parameters (*m*, *N* and *s*) in a wide range of values of interest in urban scenarios. After the analysis, we obtained the expression for *f*(*m*, *N*, *s*) shown in Equation (23). Then, we used Equation (24) to estimate the collision probability of packets.

(23) $$f(m,N,s)=-7.4754\cdot 10^{-5}\cdot m-8.9836\cdot 10^{-3}\cdot N-1.4289\cdot 10^{-3}\cdot s+1.9846$$

(24) $$p(m,N,s)=f(m,N,s)\cdot p_{\text{hello}}(m,N,s)$$

Finally, the value for the available bandwidth of each neighbor, *i*, is obtained using Equation (22), where *p_m_* is now substituted by the new *p*(*m*, *N*, *s*). Then, using Equation (25), the algorithm assigns a value to the available bandwidth metric (*u*_4,_*_i_*), which is closer to one when the bandwidth is very high. Again, the use of an exponential function helps us to emphasize good nodes compared to the others.

(25) $$u_{4,i}(m,N,s)=e^{-\frac{1}{{\text{ABE}}_{i}}}$$

### 3.5. Forwarding Decision

MMMR makes forwarding decisions hop-by-hop based on geographic information. When a node wants to send a packet, it has first to find the optimal next forwarding node from its list of neighbors.

When a sender node receives hello messages from its neighbors in transmission range, the node updates its neighbors list with all those nodes that sent their packets with enough power to be considered as a neighbor. After that, the node evaluates and assigns a total multimetric qualification to each neighbor. We assign the same weights (*w*_1_, *w*_2_, *w*_3_, *w*_4_) to each metric (*u*_1_, *u*_2_, *u*_3_*, u*_4_) in the qualification of each neighbor, *i*. We propose a weighted geometric average metric with Equation (26). A global geometric score is used, because it is less sensitive than the arithmetic metric in the extreme values of the metric components. Due to the fact that all the metrics are exponential functions with the same base, the next forwarding node will be the one that has the greatest sum of all the exponents.

(26) $$\begin{array}{ll} {\bar{u}}_{i} & =\overset{4}{\underset{j=1}{\text{∏}}}u_{j}^{w_{j}}=u_{1}^{w_{1}}\cdot u_{2}^{w_{2}}\cdot u_{3}^{w_{3}}\cdot u_{4}^{w4}\\ & =\overset{4}{\underset{j=1}{\text{∏}}}u_{j}^{w_{j}}=e^{-{\left(\frac{d_{i}}{d_{\text{ref}}}\right)}^{\alpha w_{1}}}\cdot e^{-{\left(\frac{\Delta d(t)}{d_{\text{ref}}}\right)}^{\alpha w_{2}}}\cdot e^{-{\frac{1}{\rho_{i}(t)}}^{w_{3}}}\cdot e^{-{\frac{1}{{\text{ABE}}_{i}}}^{w_{4}}} \end{array}$$

Because of this, to simplify the computation of the final value, the logarithmic of each metric is used as shown in Equation (27), which is a monotonic transformation of the original score function.

(27) $$\begin{array}{cc} \ln{\bar{u}}_{i} & =\overset{4}{\underset{j=1}{\text{∑}}}w_{j}\cdot \ln u_{j}=w_{1}\cdot \ln e^{-{\left(\frac{d_{i}}{d_{\text{ref}}}\right)}^{\alpha }}+w_{2}\cdot \ln e^{-{\left(\frac{\Delta d(t)}{d_{\text{ref}}}\right)}^{\alpha }}+w_{3}\cdot \ln e^{-\frac{1}{\rho_{i}(t)}}+w_{4}\cdot \ln e^{-\frac{1}{{\text{ABE}}_{i}}}\\ & =-w_{1}\cdot {\left(\frac{d_{i}}{d_{\text{ref}}}\right)}^{\alpha }-w_{2}\cdot {\left(\frac{\Delta d(t)}{d_{\text{ref}}}\right)}^{\alpha }-w_{3}\cdot -\frac{1}{\rho_{i}(t)}-w_{4}\cdot \frac{1}{{\text{ABE}}_{i}}\\ -\ln{\bar{u}}_{i} & =w_{1}\cdot {\left(\frac{d_{i}}{d_{\text{ref}}}\right)}^{\alpha }+w_{2}\cdot \left(\frac{\alpha \cdot d^{\alpha -1}\cdot \left\|\vec{v}\right\|\cdot \cos\theta }{d_{\text{ref}}^{\alpha }}\right)+w_{3}\cdot \frac{1}{\rho_{i}(t)}+w_{4}\cdot \frac{1}{{\text{ABE}}_{i}} \end{array}$$

We give a score to each node using the negative of the logarithm in the final calculation of the metric. Therefore, the best next forwarding node is the neighbor with the lowest multimetric value obtained applying the MMMR algorithm. Notice that the exponent of the metrics distance and trajectory include also the exponent alpha, which could be merged with the weight of the metric (see Equation (26)) in a unique exponent as a new compound weight. Nevertheless, we preferred not to do that in order to emphasize the difference between the metric itself and the correspondent weight and to not distort their role in the equation. As previously said, we give the same degree of importance to all the metrics, *i.e.*, we set *w_j_* = 1/4, as the starting point of our work.

## 4. Simulation Results

### 4.1. Simulation Scenario

We analyzed the performance of our multimetric algorithm MMMR and compared it to AODV [19], GPSR [6], I-GPSR [11] and GBSR-B [20]. To do this, we carried out several simulations using the NCTUns 6.0 (National Chiao Tung University network simulator) [21]. We used the NCTUns simulator due to the fact that it allows for a simple and accurate way of designing VANET scenarios [22]. It is possible to use our own mobility model, including walls to attenuate the signal, among others.

GBSR-B is our previous proposal that improves GPSR with its map-aware capability and a buffer scheme to be used instead of the perimeter mode of GPSR, as was explained in Section 3.2. GBSR-B only uses the distance as the metric to make forwarding decisions.

**Figure 3.**
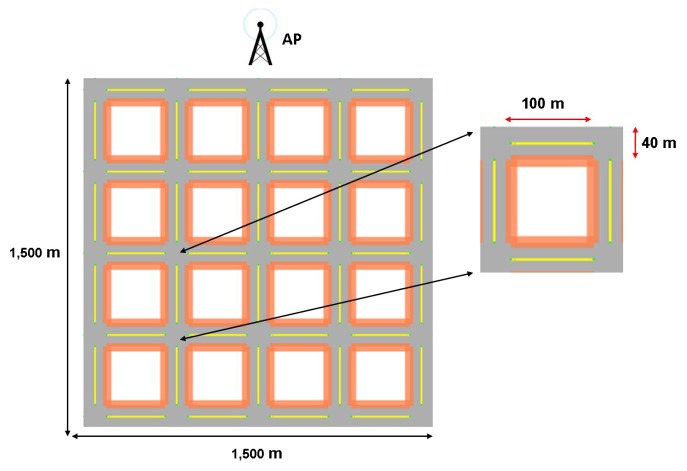
Dimensions of the streets in the urban scenario used in the evaluations and localization of the access point (AP; destination).

We used a grid scenario to model a common urban scenario formed by streets and crossroads. Seeking to simulate a realistic scenario, we used Citymob [23] to generate the movements of vehicles that follow streets and respect the presence of other vehicles and traffic lights. We use in the scenarios blocks (orange lines) that work as buildings. These walls are set with the total block of the signal, and the simulation process of attenuation is detailed in [24]. The simulation area was 1,500 m × 1,500 m. Each street was 100 m-long, with intersections of 40 m (see Figure 3) according to the area of the example in Barcelona, Spain. We considered two densities of vehicles (60 and 120 vehicles), which were randomly positioned. The maximum average speed of the vehicles was 50 km/h. There was one fixed destination, the access point (henceforth, called AP; see Figure 3), through which vehicles connect to the network to report traffic information. We use a fixed AP position in the border of the scenario, because in this way, we obtained different route lengths, depending on the position of the cars in the scenario. Half of the nodes sent 1,000-byte packets every 2 s to the unique destination for 1,000 s. The traffic profile is CBR (Constant Bit Rate) 4 Kbps per source. There is an interfered traffic of 800 Kbps. The simulations were carried out using the IEEE 802.11 p standard on physical and MAC layers. The latter was simulated with only the best effort (BE) access category. Furthermore, we use the two-ray ground as the radio propagation model joint to the Rician fading model. We set an average transmission range of 250 m, which is a typical parameter in vehicular environments. All the figures are presented with confidence intervals (CI) of 95% obtained from five simulations per point. Table 4 summarizes the main simulation settings.

### 4.2. Comparison between MMMR and Other Routing Protocols

In this section, we present the results of the evaluation of MMMR in two different scenarios: a low density scenario with 60 nodes and a medium density scenario with 120 nodes. A medium density scenario in this case is enough to evaluate the protocol routing proposed without causing medium saturation. Each one is analyzed using different routing protocols to see the benefits that MMMR offers. The well-known routing protocols, AODV [19] and GPSR [6], are evaluated as references. We show the results with AODV for comparison purposes, because this protocol is an important reference in mobile *ad hoc* networks and is the basis for other reactive protocols designed for VANETs. We implemented another proposal, named I-GPSR [11], because it has some similarities with our proposal. Furthermore, we use our previous proposal, called GBSR-B [20], which includes a buffer to temporarily store packets instead of dropping them when there is no proper next hop. GBSR-B is also building-aware to avoid sending packets to nodes in transmission range that are behind a building. Finally, we simulate MMMR configured with a weight of 0.25 in each metric (see Equation (27)).

**Figure 4.**
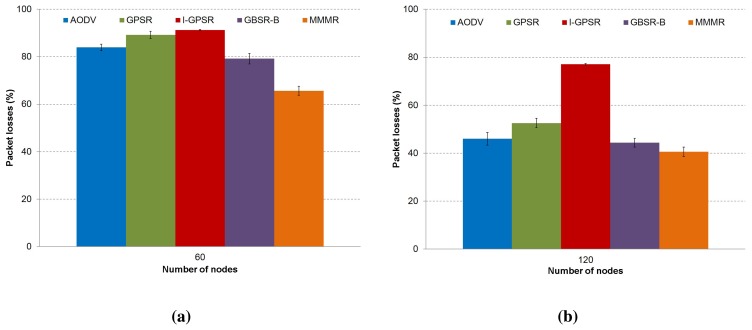
The behavior of the protocols AODV, GPSR, I-GPSR, GBSR-B and MMMR in terms of the percentage of packet losses are presented in (**a**) a low density scenario (60 nodes) and (**b**) a medium density scenario (120 nodes) (CI 95%).

First, we can see in Figure 4 the percentage of packet losses obtained using each one of the evaluated protocols. We can notice that MMMR obtains the best results in both scenarios. This is due to the optimal selection of the next forwarding node based on the four proposed metrics. Besides, GBSR-B achieves a good result, due to the use of the buffer as an alternative to the perimeter mode used in GPSR, which results in lower packet losses. We observe lower packet losses in the medium density scenario. This makes sense, since the presence of a higher number of neighbors allows for a better selection of the next forwarding node. Regarding AODV, it is known that AODV is not an efficient routing protocol for VANETs, because it establishes a full end-to-end path. This is not suitable for VANETs, due to the high mobility of the nodes. When the link is established, however, it can successfully send a high number of packets; see Figure 4a,b.

Meanwhile, GPSR uses the perimeter mode (as a recovery path process) that is not very efficient and produces a considerable number of packet losses. Finally, we see that the highest number of packet losses is obtained by I-GPSR in both low and medium density scenarios. This behavior of I-GPSR is mainly due to how the selection of the next hop is designed. The forwarding operation of I-GPSR is based on distance, speed, moving direction and density, which are adequate metrics for VANETs. I-GPSR prefers those vehicles that approach the destination faster, which is good in VANETs. Nevertheless, since our urban scenario has a mobility model that includes stops (in crossroads), it may happen that sometimes, vehicles remain static (e.g., in a crossroads, due to a traffic light), affecting the calculation of its score. I-GPSR thinks that such a stopped node is not a good forwarder node. We tackled this issue in MMMR, so that our proposed metric of the trajectory analyses the movement of the vehicle from two consecutive time instants.

**Figure 5.**
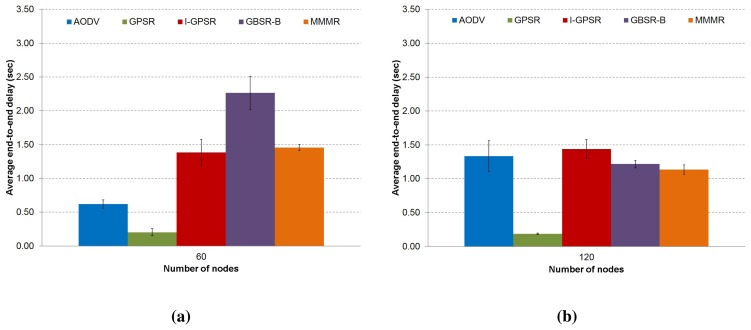
The average end-to-end packet delay in (**a**) a low density scenario (60 nodes) and (**b**) a medium density scenario (120 nodes) are presented, comparing the protocols, AODV, GPSR, I-GPSR, GBSR-B and MMMR (CI 95%).

Figure 5 shows the results of the average packet delay. The delay is calculated based on those packets that successfully arrived at the destination. Since GPSR takes the forwarding decision considering only distance, it frequently uses the perimeter mode (recovery process), which is not much more efficient and increases the probability of packet losses. GPSR obtains the lowest packet delay in both scenarios, as the second columns in Figure 5a,b shown.

This is because with GPSR, a low number of packets considered to compute the average delay, arrived at the destination. Moreover, much of the lost packets traveled through several hops before being dropped. GBSR-B obtains the highest delay in the low density scenario (see Figure 5a), due to the high amount of time that packets spend in the local buffer. This does not happen so frequently in the medium density scenario, due to the presence of a higher number of neighbors, which allows for a faster selection of an optimal next forwarding hop. The same happens with MMMR, but with better results, since it also includes the building-aware capability and the improvement of the forwarding decision with the use of the four metrics described in the previous section. In the case of AODV in the low density scenario (see Figure 5a), packets that needed a recovery process (after route breakage, new RREQ/RREP (Route Request/Route Reply) messages to find a new end-to-end path are exchanged) suffered a high loss probability and often did not arrive at the destination. Consequently, AODV obtains a low packet delay computed only from packets successfully delivered. In contrast, in the medium density scenario (see Figure 5b), packets that used the recovery process had a higher probability to arrive at the destination, due to the presence of a high number of neighbors, which offers more options to forward the packets. However, those packets suffered a higher packet delay (Figure 5b). Finally, we can see that I-GPSR obtains a slightly lower delay in the low density scenario compared to MMMR, due to the high number of packet losses that used a long path, so that they were not considered in the computation of the average delay. In the medium density scenario, I-GPSR obtains the highest delay compared to the other protocols, due to the process of the next forwarding selection, which penalizes vehicles stopped momentarily in crossroads, although they could be good forwarding candidates.

**Figure 6.**
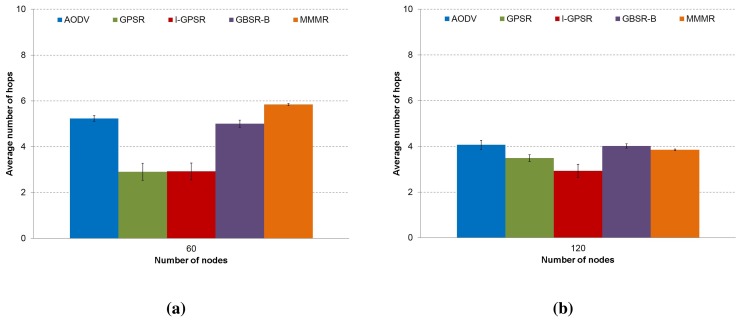
The average number of hops to the destination in (**a**) a low density scenario (60 nodes) and (**b**) a medium density scenario (120 nodes) are presented, comparing the protocols, AODV, GPSR, I-GPSR, GBSR-B and MMMR (CI 95%).

Regarding the average number of hops, depicted in Figure 6, the results are very much related to the scheme to choose the next forwarding node used by each routing protocol. We can see that GBSR-B (fourth column) delivers more packets successfully after using more hops compared to GPSR (second column). This is mainly due to the building-aware scheme of GBSR-B, which refrains from choosing vehicles behind buildings, which reduces the losses (see Figure 4), although more hops are used. In AODV (first column), we can see that in the medium density scenario (Figure 6b), there are paths with a lower number of hops compared to the low density scenario (Figure 6a), since shorter paths could be formed among more nodes to be chosen. However, AODV spends more time in establishing the full path, obtaining, therefore, a high delay (see Figure 5b). Notice that our MMMR presents the highest number of hops in the low density scenario (Figure 6a). The reason is that MMMR not only uses distance as the metric to choose the next forwarding nodes; MMMR also uses the trajectory, density and available bandwidth that longer number of hops will use. In the medium density scenario (Figure 6b), a lower number of hops were needed, since there are more neighbors to choose a proper next hop according to the four-metric algorithm.

Notice that our proposal is mainly focused on VANETs, and we try to increase the number of packets that arrive at the destination. With our proposal, packets sent from farther distances, which need more hops to get to the destination, can reach the destination more frequently, thus increasing the average number of hops. Besides, the high number of hops is not a drawback from the power consumption point of view, since in VANETs (contrary to WSN or MANETs), the on-board unit (OBU) of the vehicle is powered by the car battery. Hence, the communication equipment do not have battery constraint problems.

**Figure 7.**
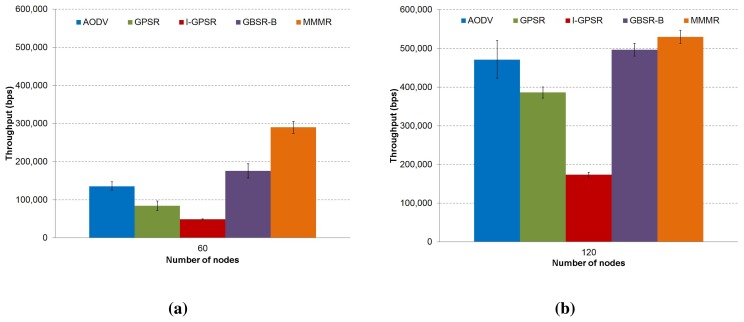
The total throughput of the received packets comparing the protocols, AODV, GPSR, I-GPSR, GBSR-B and MMMR, are presented in (**a**) a low density scenario (60 nodes) and (**b**) a medium density scenario (120 nodes) (CI 95%).

Finally, Figure 7 shows the throughput achieved using each protocol. Clearly, protocols with high packet losses (I-GPSR in Figure 4) obtain a low throughput (I-GPSR in Figure 7). In contrast, low packet losses (MMMR in Figure 4) represent a high throughput (MMMR in Figure 7).

The results confirm that the use of a four-metric combination used in our proposal to score candidates improves the selection of nodes in different environments compared to other routing protocols proposed for VANETs.

### 4.3. Performance Evaluation of MMMR

**Figure 8.**
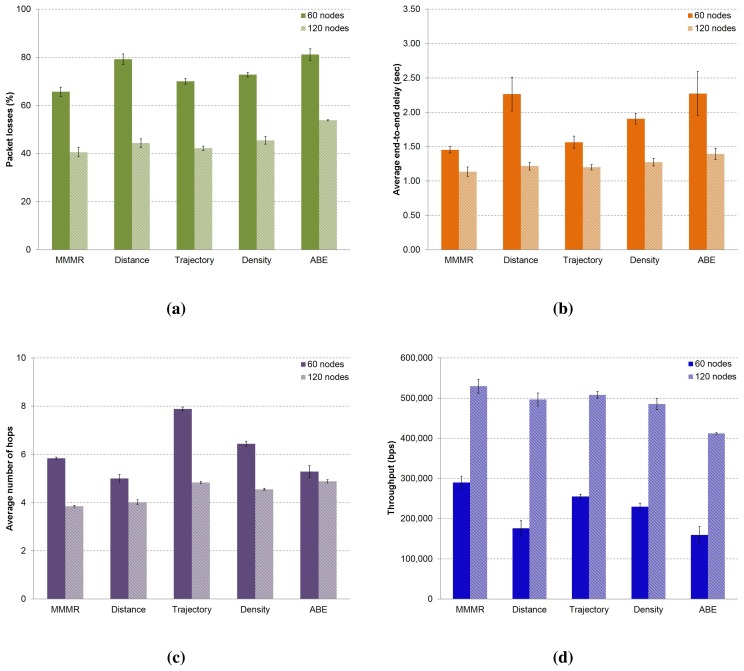
The performance of multimetric routing using single metrics in a low (60 nodes) and a medium (120 nodes) density scenario compared to MMMR. The results show: (**a**) the percentage of packet losses; (**b**) the average packet delay; (**c**) the average number of hops to destination; and (d) the throughput of the received packets (CI 95%).

In this section, we present a performance evaluation of the MMMR protocol using the same weight in each one of the four metrics, *i.e.*, *w_j_* = 1/4. We compare the results to a modified MMMR in which routing decisions are made considering only one single metric. The evaluation was made in both low and medium density scenarios, and the results are depicted in Figure 8.

We can see that MMMR (first column) obtains the best results in terms of packet losses (see Figure 8a), followed by the option of taking the forwarding decision based only on the metric “trajectory”, that is, (*w*_1_, *w*_2_, *w*_3_, *w*_4_) = (0, 1, 0, 0). Packet losses (Figure 8a), end-to-end delay (Figure 8b) and the average number of hops (Figure 8c) are best when the density of the scenario is 120 nodes, because the probability of finding a good neighbor is higher than in the case of low density scenarios (60 nodes). Moreover, in the low density scenario, the probability of using the buffer to store packets is higher, so that higher delays are obtained. Given that throughput is related to packet losses, we can see that in the medium density scenario, there is a higher throughput (Figure 8d), because lower losses were produced (Figure 8a).

With these results, we can conclude that our proposal based on the use of four metrics to make forwarding decisions is a good choice to decrease the packet losses in vehicular scenarios and to obtain a low delay in both low and medium density scenarios.

**Figure 9.**
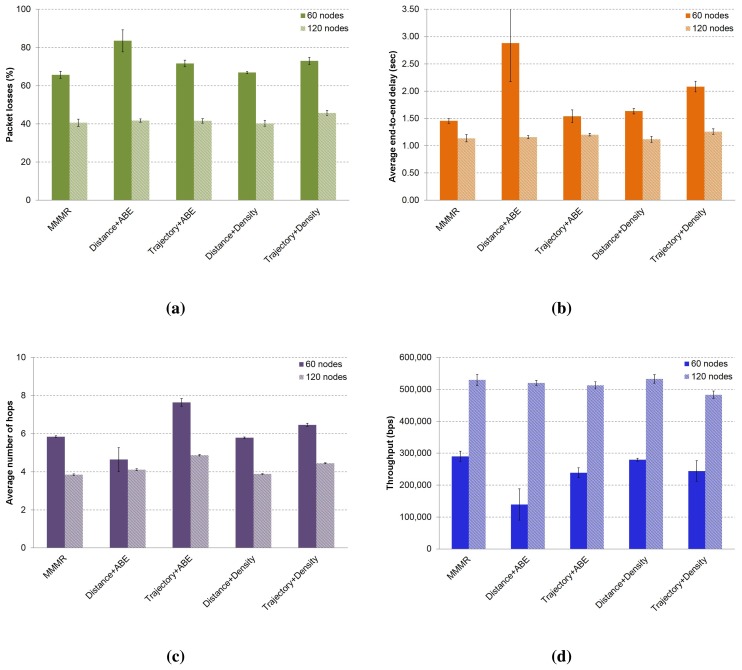
The performance of MMMR using four combination of metrics in low (60 nodes) and medium (120 nodes) density scenarios, compared to MMMR using the four metrics. The results show: (**a**) the percentage of packet losses; (**b**) the average packet delay; (c) the average number of hops to the destination; and (**d**) the throughput of the received packets (CI 95%).

In Figure 9, we present the results of the MMMR protocol compared to another modified version that considers only some combinations of metrics. Packet losses are slightly lower in the medium density scenario when forwarding decisions are taken based on the combination of the metric “distance + density”, (*w*_1_, *w*_2_, *w*_3_, *w*_4_) = (0.5, 0, 0.5, 0). However, it is not the case in the low density scenario, where the best results are obtained with MMMR (*w*_1_, *w*_2_, *w*_3_, *w*_4_) = (0.25, 0.25, 0.25, 0.25); see Figure 9a. These results prove that the density of the scenario is a very important parameter in VANETs to obtain the final score of the candidates to make the forwarding decisions. The behavior of packet delay and the number of hops in the low density scenario are as expected, *i.e.*, a low number of hops produces a high delay, as is the case for the “distance + ABE” combination (*w*_1_, *w*_2_, *w*_3_, *w*_4_) = (0.5, 0, 0, 0.5). The reason is that the use of the buffer, i.e., short paths in low density scenarios, means that packets spent a significant time in the buffer. In the case of the medium density scenario, it shows a quite stable number of hops and lower delays, because there is a higher number of neighbors, and it was not necessary to use the local buffer of the nodes often to store packets temporarily until finding a good next forwarder; see Figure 9a,b.

We can conclude that the metric of the vehicles' density is decisive in both scenarios, since it has a high impact on the results. Furthermore, we can notice that it is possible to have good metric combinations that improve packet losses and delay, as is the case for “distance + density” (*w*_1_, *w*_2_, *w*_3_, *w*_4_) = (0.5, 0, 0.5, 0) in the medium density scenario.

In Tables 5 and 6, we summarize some combination of the weights in the low and medium density scenarios, respectively. Both tables show the percentage of losses and the average delay obtained with each combination of weights.

We can see in Table 5 that the best results, in terms of packet losses and average delay, are obtained with the MMMR combination of weights (*w*_1_, *w*_2_,*w*_3_, *w*_4_) = (0.25, 0.25, 0.25, 0.25). In the case of the medium density scenario (see Table 6), the best option is the combination “distance + density” (*w*_1_, *w*_2_, *w*_3_, *w*_4_) = (0.5, 0, 0.5, 0). The second best option is our proposal (*w*_1_, *w*_2_, *w*_3_, *w*_4_) = (0.25, 0.25, 0.25, 0.25), which, differently from the “distance + density” option, includes a bandwidth guarantee by using the ABE algorithm.

The possible drawback of MMMR is the simple way of assigning weights to each metric, i.e., equitably.

## 5. Conclusions and Future Work

In this paper, we presented a new routing protocol for VANETs that includes four metrics (distance, trajectory, density and ABE) to make hop-by-hop forwarding decisions. Furthermore, the proposal is building-aware, avoiding those nodes in transmission range, but behind a building. This feature protects packets from being thrown out. Besides, a local buffer is used to temporarily store those packets when the routing protocol fails in finding a proper next forwarding node. We analyzed several metrics and developed an algorithm that includes four of them. The algorithm computes a global score value used to select the best next forwarding node among all the neighbors in transmission range.

Firstly, we evaluated our proposal compared to other routing proposals present in the literature in two scenarios (a low and a medium density scenario). In terms of packets losses and throughput, MMMR improves all the protocols evaluated, due to the new way of selecting the next forwarding node. Compared to other existing routing protocols, we concluded that MMMR has a better performance in both scenarios, low and medium density.

Secondly, we presented a performance evaluation, taking into account the forwarding decision based on each single metric compared to MMMR. The results show that MMMR, which makes the next forwarding decision using the equitable combination of the four metrics, is better than making the forwarding decision based only on one of the four metrics independently.

Finally, we evaluated some combinations of the four metrics to analyze the behavior depending on the scenario. In this evaluation, we noticed that in the case of the 60 node scenario, the best results were obtained with MMMR. In terms of packet losses, MMMR was followed by the combination of weights *distance*+*density*, while, in terms of average delay, by the combination of weights *ABE* +*trajectory*. This makes sense, since in scenarios with a low density of vehicles, it is better to select nodes that are moving towards the destination. In the case of the 120 node scenario, the best results are obtained by the distance + density combination, followed in second place by MMMR.

As future work, we plan to analyze other methods to obtain a global metric value. Furthermore, the computation of the weights of the metrics will be improved in a future work using machine learning techniques [25] to auto-configure the weights, varying throughout time, making the algorithm adaptable to the changing network conditions that are inherent in VANETs. Finally, we also will explore the available power level of each node as a metric to mark nodes as reliable next forwarding nodes.

## Figures and Tables

**Table 1.** Hello messages (HMs) format in the multimetric map-aware routing protocol.

**ID**	***l****_x_*	***l****_y_*	***v****_x_*	***v****_y_*	***S***	***t*_*idle*_**	***ρ***
32 bits	32 bits	32 bits	8 bits	8 bits	16 bits	8 bits	8 bits

**Table 2.** Extra data per node used in the neighbor list included in the HM.

**Neighbor *i***	**Reception Time of the Last HM**	**Reception Time of the First HM**	**No. HM**
32 bits	8 bits	8 bits	8 bits

**Table 3.** The main simulation setting used in the distance evaluation

**Parameter**	**Value**
Simulation area	1,500 m × 1,500 m
Number of nodes	120 vehicles
Maximum node speed	50 km/h
Transmission/sensing range	250/300 m
MAC specification	IEEE 802.11p
QoS access category	BE (best effort)
Bandwidth	12 Mbps
Simulation time	1,000 s
Maximum packet size	1,000 bytes
Traffic profile	CBR (Constant Bit Rate) 4 Kbps
Routing protocol	GPSR

**Table 4.** The main simulation settings used in the evaluation scenarios.

**Parameter**	**Value**
Simulation area	1,500 m × 1,500 m
Number of nodes	60 and 120 vehicles
Maximum node speed	50 km/h
Transmission range	250 m
Sensing range	300 m
Mobility model	Double-Manhattan
Downtown area	700 × 700 m
Mobility generator	Citymob
MAC specification	IEEE 802.11p
QoS access category	BE (best effort)
Bandwidth	12 Mbps
Simulation time	1,000 s
Maximum packet size	1,000 bytes
Traffic profile	CBR 4 Kbps
Routing protocol	AODV, GPSR, GBSR-B, I-GPSR, MMMR

**Table 5.** A summary of the results of the evaluation of some combination of the weights for each metric (in a low density scenario). ABE, available bandwidth estimator.

**Distance** ***w*_1_**	**Trajectory** ***w*_2_**	**Density** ***w*_3_**	**ABE** ***w*_4_**	**% Losses**	**Average Delay** **[s]**
**0.25**	**0.25**	**0.25**	**0.25**	**65.66**	**1.45**
1	0	0	0	79.17	2.26
0	1	0	0	70.02	1.56
0	0	1	0	72.79	1.91
0	0	0	1	81.14	2.27
0.5	0	0	0.5	83.50	2.88
0	0.5	0	0.5	71.62	1.54
0.5	0	0.5	0	66.90	1.63
0	0.5	0.5	0	72.98	2.08

**Table 6.** A summary of the results of the evaluation of some combination of the weights for each metric (in a medium density scenario).

**Distance** ***w***_1_	**Trajectory** ***w***_2_	**Density** ***w***_3_	**ABE** ***w***_4_	**% Losses**	**Average Delay** **[s]**
0.25	0.25	0.25	0.25	40.58	1.14
1	0	0	0	44.36	1.22
0	1	0	0	42.13	1.20
0	0	1	0	45.47	1.27
0	0	0	1	53.81	1.40
0.5	0	0	0.5	41.75	1.16
0	0.5	0	0.5	41.56	1.20
**0.5**	**0**	**0.5**	**0**	**40.29**	**1.12**
0	0.5	0.5	0	45.75	1.26
